# Dectin-2-Targeted Antifungal Liposomes Exhibit Enhanced Efficacy

**DOI:** 10.1128/mSphere.00715-19

**Published:** 2019-10-30

**Authors:** Suresh Ambati, Emma C. Ellis, Jianfeng Lin, Xiaorong Lin, Zachary A. Lewis, Richard B. Meagher

**Affiliations:** a Department of Genetics, University of Georgia, Athens, Georgia, USA; b Department of Microbiology, University of Georgia, Athens, Georgia, USA; Duke University Medical Center

**Keywords:** liposomes, Dectin-2, exopolysaccharide matrix, mannan, amphotericin B, invasive fungal disease

## Abstract

Candida albicans, Cryptococcus neoformans, and Aspergillus fumigatus cause life-threatening candidiasis, cryptococcosis, and aspergillosis, resulting in several hundred thousand deaths annually. The patients at the greatest risk of developing these life-threatening invasive fungal infections have weakened immune systems. The vulnerable population is increasing due to rising numbers of immunocompromised individuals as a result of HIV infection or immunosuppressed individuals receiving anticancer therapies and/or stem cell or organ transplants. While patients are treated with antifungals such as amphotericin B, all antifungals have serious limitations due to lack of sufficient fungicidal effect and/or host toxicity. Even with treatment, 1-year survival rates are low. We explored methods of increasing drug effectiveness by designing fungicide-loaded liposomes specifically targeted to fungal cells. Most pathogenic fungi are encased in cell walls and exopolysaccharide matrices rich in mannans. Dectin-2 is a mammalian innate immune membrane receptor that binds as a dimer to mannans and signals fungal infection. We coated amphotericin-loaded liposomes with monomers of Dectin-2’s mannan-binding domain, sDectin-2. sDectin monomers were free to float in the lipid membrane and form dimers that bind mannan substrates. sDectin-2-coated liposomes bound orders of magnitude more efficiently to the extracellular matrices of several developmental stages of C. albicans, C. neoformans, and A. fumigatus than untargeted control liposomes. Dectin-2-coated amphotericin B-loaded liposomes reduced the growth and viability of all three species more than an order of magnitude more efficiently than untargeted control liposomes and dramatically decreased the effective dose. Future efforts focus on examining pan-antifungal targeted liposomal drugs in animal models of fungal diseases.

**IMPORTANCE** Invasive fungal diseases caused by Candida albicans, Cryptococcus neoformans, and Aspergillus fumigatus have mortality rates ranging from 10 to 95%. Individual patient costs may exceed $100,000 in the United States. All antifungals in current use have serious limitations due to host toxicity and/or insufficient fungal cell killing that results in recurrent infections. Few new antifungal drugs have been introduced in the last 2 decades. Hence, there is a critical need for improved antifungal therapeutics. By targeting antifungal-loaded liposomes to α-mannans in the extracellular matrices secreted by these fungi, we dramatically reduced the effective dose of drug. Dectin-2-coated liposomes loaded with amphotericin B bound 50- to 150-fold more strongly to C. albicans, C. neoformans, and A. fumigatus than untargeted liposomes and killed these fungi more than an order of magnitude more efficiently. Targeting drug-loaded liposomes specifically to fungal cells has the potential to greatly enhance the efficacy of most antifungal drugs.

## Abstract (precis)

Invasive fungal diseases caused by Candida albicans, Cryptococcus neoformans, and Aspergillus fumigatus have mortality rates ranging from 10 to 95%. Individual patient costs may exceed $100,000 in the United States. All antifungals in current use have serious limitations due to host toxicity and/or insufficient fungal cell killing that results in recurrent infections. Few new antifungal drugs have been introduced in the last 2 decades. Hence, there is a critical need for improved antifungal therapeutics. By targeting antifungal-loaded liposomes to α-mannans in the extracellular matrices secreted by these fungi, we dramatically reduced the effective dose of drug. Dectin-2-coated liposomes loaded with amphotericin B bound 50- to 150-fold more strongly to C. albicans, C. neoformans, and A. fumigatus than untargeted liposomes and killed these fungi more than an order of magnitude more efficiently. Targeting drug-loaded liposomes specifically to fungal cells has the potential to greatly enhance the efficacy of most antifungal drugs.

## INTRODUCTION

Candida albicans is a commensal fungus found on the skin and in the intestinal track, while Cryptococcus neoformans and Aspergillus fumigatus are indigenous to soil. Candidiasis, cryptococcal meningitis, and pulmonary aspergillosis claim more than one million lives annually (1–5). Health care costs in the United States for these three invasive fungal diseases exceed 4.7 billion dollars annually (6). Patients at the greatest risk of developing life-threatening fungal infections include those with weakened immune systems and/or various lung diseases, such as immunosuppressed and immunocompromised individuals, patients receiving long-term treatment for inflammatory diseases, and AIDS patients. Furthermore, the number of immunocompromised individuals, who are susceptible to these opportunistic fungal infections, is increasing due to growing numbers of patients on immunosuppressants as part of their therapy for cancer, for stem cell and organ transplants, and for inflammatory diseases (3, 7).

Patients with candidiasis, cryptococcosis, or aspergillosis are treated with antifungals such as the fungicidal polyene amphotericin B (AmB) and/or other fungicides. However, even with antifungal therapies, there are alarmingly high mortality rates that range from 10% to 95%, depending upon the patient population and particular fungal infection (1–5, 8, 9). Furthermore, all known synthetic antifungal agents such as AmB are toxic to human cells and organs. For example, AmB treatment frequently causes renal toxicity that is often fatal (10–12), and diverse prophylactic strategies are employed to reduce adverse outcomes for patients (13). The incidence of antifungal-resistant *Cryptococcus* and *Candida species* is increasing (14–18). There are few new antifungal drugs in play (19). Of the ten antifungals listed by the CDC (2) as commonly employed to treat these diseases, only isavuconazole was introduced in the last 2 decades (20, 21). Clearly, there is a need for new antifungal therapeutic treatments with improved efficacy.

Since the 1980s, antibodies have been used to target anticancer drug-loaded liposomes to cancer cells with demonstrated improvements in therapeutic efficiency (22, 23). Antibody-coated immunoliposomes target the delivery of bundled anticancer drugs specifically to tumors or dispersed cancer cells compared to the passive delivery of drugs to all organs and cells by untargeted drug-loaded liposomes or free drug. Antibody-targeted drug-loaded liposomes generally increase cell-type specificity and reduce host cytotoxicity by 5- to 10-fold over passive delivery (24–26). Several targeted liposomal drugs have been FDA approved (27–31). Our goal has been to target antifungals packaged in liposomes specifically to fungal cells. The pharmacokinetic concept behind the improved performance of targeted lipsomes is that by maintaining high drug concentration in the cell vicinity, the efficacy of drug delviery is increased, while the dose delivered to the patient is reduced, and hence, host toxicity is reduced.

As with many pathogenic fungi, the outer layer of the cell walls and extracellular matrices of C. albicans, C. neoformans, and A. fumigatus and the extracellular capsule of C. neoformans contain mannan-rich polysaccharides and mannoproteins. Their extracellular matrices are essential for these pathogenic fungal cells to adhere to host cell and organ surfaces and to implanted medical devices (32–36) and confer increased resistance to antifungals (37, 38). Hence, the extracellular matrix contributes to poor clinical outcomes (33, 34, 39–41). Herein, we sought to target fungicide-loaded liposomes specifically to mannans, thereby increasing drug concentration at or near to the fungal cell surface and improving antifungal therapeutic efficiency.

Dectin-2 is encoded by the human and mouse C-type LECtin receptor gene *CLEC6A.* Dectin-2 binds α-mannans and N-linked and O-linked mannans in mannoproteins (42–46). Dectin-2 is expressed in the plasma membrane of some lymphocytes, with its mannan binding domain (sDectin-2) on the outside of these cells and its signaling domain in the cytoplasm. Dectin-2 functions as an innate immune receptor that signals the host of an active fungal infection (45, 47–50).

We recently constructed Dectin-1-coated amphotericin B (AmB)-loaded liposomes and showed these are targeted to the β-glucans in the inner cell walls and bind efficiently to multiple cell types of Aspergillus fumigatus and to C. neoformans yeast cells. Dectin-1-coated AmB-loaded liposomes (DEC1-AmB-LLs) efficiently inhibit and kill A. fumigatus cells (51). However, Dectin-1-coated liposomes bind poorly to C. albicans, presumably due to the presence of a thick mannan polysaccharide and mannoprotein outer layers masking their β-glucans (52–56). Herein, we coated AmB-loaded liposomes with the mannan-binding domain of mouse Dectin-2, sDectin-2. The sDectin-2-coated AmB-loaded liposomes bound efficiently to C. albicans, C. neoformans, and A. fumigatus and dramatically reduced cell growth and viability relative to untargeted drug-loaded liposomes.

## RESULTS

### Preparation of fungicide-loaded sDectin-2-coated fluorescent liposomes.

A model of the fungicide-loaded, sDectin-2-coated liposomes constructed herein is shown in Fig. S1 in the supplemental material. Liposome construction methods and liposome composition paralleled closely those of sDectin-1-coated liposomes described recently (51). We remotely loaded 11 mol% amphotericin B (AmB) relative to moles of liposomal lipids into the membrane of pegylated liposomes to make AmB-loaded liposomes (AmB-LLs) (51). For reference, the widely used commercial AmB-loaded untargeted liposomal product AmBisome contains 10.6 mol% AmB relative to moles of liposomal lipid (57–59) (see Table S1 in the supplemental material). We designed the murine sDectin-2 sequence to contain a small lysine tag on its amino-terminal end (see Fig. S2 in the supplemental material), expressed it in Escherichia coli (see Fig. S3 in the supplemental material), and conjugated the purified sDectin-2 protein via this lysine tag to NHS-PEG-DSPE (*N*-hydroxysuccinimide plus polyethylene glycol plus 1,2-distearoyl-*sn*-glycero-3-phosphoethanolamine), making DEC2-PEG-DSPE. We then incorporated DEC2-PEG-DSPE via its DSPE lipid moiety into AmB-LLs at 1 mol% protein molecules relative to moles of liposomal lipid (1,500 molecules of sDectin-2 per liposome) to make DEC2-AmB-LLs. Similarly, and as a protein-coated liposomal control, 0.33 mol% bovine serum albumin (BSA) was incorporated via a lipid carrier into AmB-LLs to make BSA-AmB-LLs. This resulted in equivalent microgram amounts of 22-kDa sDectin-2 and 66-kDa BSA proteins on the surface of these two sets of liposomes. Uncoated AmB-LLs, which closely resemble the commercial product AmBisome, also served as a liposomal control. Two moles percent DHPE (1,2-dihexadecanoyl-*sn*-glycero-3-phosphoethanolamine, triethylammonium salt)-rhodamine was also incorporated into the liposomal membrane of all three liposome preparations. Hence, all three sets of liposomes contained the same 11 mol% AmB and 2 mol% rhodamine. The composition of DEC2-AmB-LLs is compared to that of BSA-AmB-LLs, AmB-LLs, AmBisome, and AmB/micelles (a deoxycholate [DOC] micelle suspension delivering AmB) in Table S1.

10.1128/mSphere.00715-19.1 FIG S1 Model of sDectin-2-coated liposomes loaded with rhodamine and amphotericin B. Amphotericin B (AmB [blue ovoid structure]) was intercalated into the lipid bilayer of 100-nm-diameter liposomes. sDectin-2 (DEC2 [green globular structure]) was coupled to the lipid carrier DSPE-PEG, and both DEC2-PEG-DSPE and red fluorescent DHPE-rhodamine (red star) were inserted into the liposomal membrane via their lipid moieties, DSPE and DHPE. sDectin-2, rhodamine, AmB, and liposomal lipids are in a 1:2:11:100 mol ratio. Two sDectin-2 monomers (two DEC2-PEG-DSPE molecules) must float together in the membrane to bind to fungal mannans (red sugar moieties). From these mole ratios, the surface area of an 100-nm-diameter liposome and the published estimate of 5 × 10^6^ lipid molecules per 10^6^ nm^2^ of lipid bilayer (87), we calculated that in each of the DEC2-AmB-LLs, there are approximately 1,500 sDectin-1 monomers, 3,000 rhodamine molecules, and 16,500 AmB molecules. Download FIG S1, JPG file, 0.5 MB. Copyright © 2019 Ambati et al. 2019 Ambati et al. This content is distributed under the terms of the Creative Commons Attribution 4.0 International license.

10.1128/mSphere.00715-19.2 FIG S2 The modified mouse sDectin-2 DNA *MmsDectin2lyshis* and protein MmsDectin-2. (A) DNA sequence. The codon-optimized DNA sequence of *MmsDECTIN2lyshis* was cloned into in pET-45B. NCBI BankiT no. MN104679. The vector pET-45b+ sequence of 9 codons is boxed with the start codon underlined; cloning sites KpnI (GGTACC) and PacI (TTAATTAA), respectively, are underlined. Codons for Gly, Ser (G, S) flexible linker residues are in boldface, and reactive Lys (K) residues AAG are in boldface and italic. The sDectin-2 sequence from the mouse Dectin 2 gene *CLEC6A*, which was codon optimized for E. coli expression, is in plain text, ending in an Ala (A) codon, GCT, and two stop codons, TAA and TTA, in boldface within the PacI site. The length of the coding sequence and two stop codons is 574 bp, and the sequence encodes a 189-residue protein. The alternative gene name is *MmsDectin2lyshis*. (B) sDectin-2 (DEC2) protein synthesized in E. coli. N-terminal amino acid peptide sequence and His_6_ (HHHHHH) affinity tag from pET-45B+ boxed, Gly Ser (GS) flexible linker residues and reactive Lys (K) residues are in boldface, with lysines in italic. The 166 mouse sDectin-2 amino acid residues are in plain text, ending in a C-terminal Ala residue (A) in boldface. There are 189 amino acids total, with an MW of 21,763.29 g/mol with theoretical pI of 6.33. The sDectin-2 sequence represents aa residues 44 to 209 from the native mouse Dectin-2 sequence. The alternative protein name is MmsDectin2lyshis protein. Download FIG S2, JPG file, 0.7 MB. Copyright © 2019 Ambati et al. 2019 Ambati et al. This content is distributed under the terms of the Creative Commons Attribution 4.0 International license.

10.1128/mSphere.00715-19.3 FIG S3 SDS-PAGE analysis of purified sDectin-2. The crude E. coli BL21 extracts not expressing and expressing sDectin-2 and purified sDectin-2 protein were examined by SDS-PAGE on a 12% gel stained with Coomassie blue. Molecular weight markers and the approximate molecular weight of modified sDectin-2 of 22 kDa are indicated on the left. Download FIG S3, JPG file, 0.2 MB. Copyright © 2019 Ambati et al. 2019 Ambati et al. This content is distributed under the terms of the Creative Commons Attribution 4.0 International license.

10.1128/mSphere.00715-19.7 TABLE S1 Liposome and micelle compositions. Chemical compositions of liposomes and micelles discussed in the manuscript are indicated as moles percent AmB, sDectin-2 or BSA, and rhodamine, with the total amount of liposomal lipids representing 100 mol%. Download Table S1, XLSX file, 0.01 MB. Copyright © 2019 Ambati et al. 2019 Ambati et al. This content is distributed under the terms of the Creative Commons Attribution 4.0 International license.

### sDectin-2-coated DEC2-AmB-LLs bound more strongly to diverse fungal species than control liposomes.

**FIG 1:**
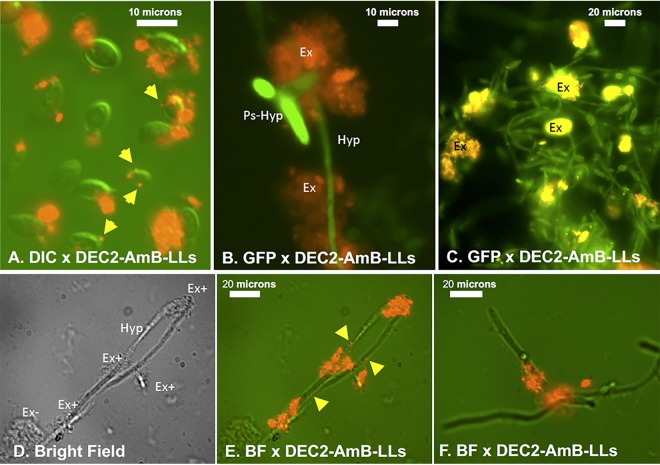
sDectin-2-coated liposomes (DEC2-AmB-LLs) bound to the extracellular matrix associated with C. albicans cells of diverse morphologies. (A) Yeast cells. Cells are highlighted by differential interference contrast (DIC) microscopy (green), and rhodamine fluorescently labeled DEC2-AmB-LLs are in red. (B and C) Pseudohyphae (Ps-Hyp) and hyphae (Hyp). Cells are highlighted via their endogenous GFP fluorescence. DEC2-AmB-LLs bound in large clusters to the extracellular matrix (Ex). (D to F). Mature hyphae. (D) Bright-field microscopy showing extracellular matrix surrounding hyphae of stained cells in panel E. (E) Combined bright-field image and red fluorescence of liposomes. (F) Additional image parallel to that in panel E reiterating a typical staining of the matrix. All cells were stained for 1 h with a 1:200 dilution of DEC2-AmB-LLs into LDB2 buffer (e.g., 0.5 μg sDectin-2/100 μl). The extracellular matrices clearly stained (Ex^+^) or weakly or not stained (Ex^−^) with DEC2-AmB-LLs are indicated. Yellow arrows indicate individual liposomes. Photographs were taken at ×63 magnification under oil immersion. Several independent fungal cell labeling studies gave similar images.

sDectin-2-coated, red fluorescent, DEC2-AmB-LLs bound strongly to C. albicans yeast cells, pseudohyphae, and hyphae (Fig. 1). The vast majority of sDectin-2-coated liposomes bound in large clusters to the extracellular polysaccharide matrix associated with these cells. Furthermore, DEC2-AmB-LLs bound to a large subset of the extracellular matrix (Ex^+^) surrounding these cells, while some regions of the matrix clearly did not bind sDectin-2-coated liposomes (Ex^−^) (Fig. 1E). Although 100-nm liposomes are too small to be resolved by light microscopy, the presence of an estimated 3,000 rhodamine molecules per liposome (Fig. S1) allows the fluorescent signal from individual liposomes to be visualized (51). We only rarely saw individual DEC2-AmB-LLs (yellow arrows in Fig. 1A) or liposome clusters binding directly to C. albicans cell walls. In contrast, we previously showed individual sDectin-1-coated liposomes bound frequently to the cell wall of A. fumigatus cells (51).

**FIG 2:**
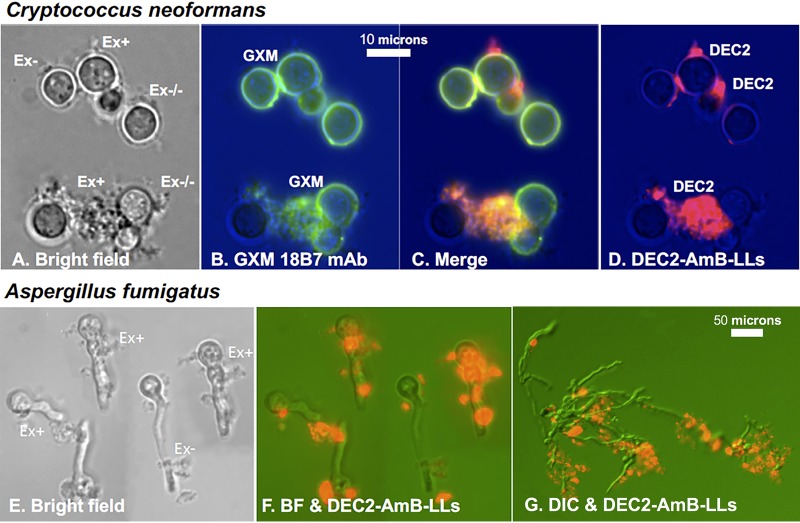
DEC2-AmB-LLs bound to the extracellular matrices associated with C. neoformans and A. fumigatus cells. Rhodamine red fluorescent DEC2-AmB-LLs bound in large clusters to the extracellular matrices (Ex) and rarely to the cell walls of both species. (A to D) C. neoformans. Yeast cells were costained with DEC2-AmB-LLs and mouse monoclonal antibody 18B7 to capsular glucuronoxylomannan (GXM) and secondary goat anti-mouse antibody Alexa 488 (green). All cells were stained for 1 h with a 1:200 dilution of DEC2-AmB-LLs into LDB2 (e.g., 0.5 μg sDectin-2/100 μl). (A) Bright-field image of cells. (B) Green fluorescent image of GXM-specific antibody-stained cells. (C) The merged fluorescent image of panels B and D. (D) Red fluorescent DEC2-AmB-LLs. (E to G) A. fumigatus. (E and F) Bright-field and combined fluorescent images of germlings grown for 10 h and stained with DEC2-AmB-LL. (G) Mature hyphae grown for 24 h. Both species were stained for 1 h with a 1:200 dilution of DEC2-AmB-LLs into LDB2 (e.g., 0.5 μg sDectin-2/100 μl). The extracellular matrices clearly stained (Ex^+^) or weakly or not stained (Ex−) with DEC2-AmB-LLs or which did not stain with either 18B7 or DEC2-AmB-LLs (Ex^−/−^) are indicated. Photographs were taken at a ×63 magnification under oil immersion (A to F) or at 20× (G). Three independent fungal cell labeling studies gave similar images.

DEC2-AmB-LLs bound strongly to C. neoformans yeast cells (Fig. 2). Monoclonal antibody 18B7 is specific for the glucuronoxylomannan (GXM) found in the capsule and exopolysaccharide of C. neoformans (60, 61). We found antibody 18B7 stained most (Fig. 2B) but not all of the cell capsules and most but not all of the exopolysaccharide that were visible in bright-field images (Ex^+^ in Fig. 2A). DEC2-AmB-LLs costained strongly most of the 18B7-stained GXM regions in the exopolysaccharide matrix, but did not stain the 187B-labeled GXM of the capsule (Fig. 2C and D). Furthermore, there were some regions of extracellular matrix that did not stain with either 18B7 or DEC2-AmB-LLs (Ex^−/−^ in Fig. 2A).

DEC2-AmB-LLs also bound in large clusters to the exopolysaccharide matrices produced by A. fumigatus germinated conidia and hyphae (Fig. 2E to G). Again, little if any binding was associated with the cell wall itself. Also, there appear to be areas where the exopolysaccharide matrix is not stained or poorly stained (Ex^−^ in Fig. 2E and F).

Because the DEC2-AmB-LLs bound poorly or not at all to mannans within the tightly cross-linked polysaccharide of cells walls of all three fungal species examined, we considered that the 100-nm-diameter size of our liposomes restricted their access or that sDectin-2 was somehow restricted from full activity when presented in a liposomal membrane. The rotational diameter of DEC2-AmB-LLs in solution would be even larger than their physical size estimate, due to their coating with sDectin-2 protein and associated water molecules. sDectin-2 itself has an atomic weight of 22 kDa and hence a rotational diameter in solution that may be estimated at ∼4 nm (62). We prepared rhodamine-coupled sDectin-2, DEC2-Rhod. The atomic weight of rhodamine (0.48 kDa) and one or two rhodamine-coupled molecules will have little effect on this size estimate for DEC2-Rhod. Red fluorescent DEC2-Rhod bound strongly to most of the exopolysaccharide matrix surrounding A. fumigatus hyphal cells (see Fig. S4A and B in the supplemental material). The pattern of binding and intensity of binding was indistinguishable from that of DEC2-AmB-LLs (Fig. S4C and D).

10.1128/mSphere.00715-19.4 FIG S4 DEC2-Rhod and DEC2-AmB-LLs bound with similar patterns to the exopolysaccharide matrices surrounding A. fumigatus hyphal cells. A. fumigatus conidia were germinated at low density on microscope chamber slides in VMM plus 1% glucose plus 0.5% BSA and grown for 24 h at 37°C and cells were fixed before staining for 1 h with rhodamine-labeled DEC2 protein or DEC2-coated liposomes. (A and B) DEC2-Rhod. (C and D) DEC2-AmB-LLs. Cells were photographed at 20×, taking differential interference contrast (DIC) images in panels A and C and combined DIC and red fluorescence images in panels B and D. Because the cells were highly dispersed and we wished to show several example cells in one plate, these images are composites made from cell images taken from separate photographic fields and placed adjacent to one another. (See the dotted outlines of cells that were moved into a common field.) Download FIG S4, JPG file, 0.3 MB. Copyright © 2019 Ambati et al. 2019 Ambati et al. This content is distributed under the terms of the Creative Commons Attribution 4.0 International license.

**FIG 3:**
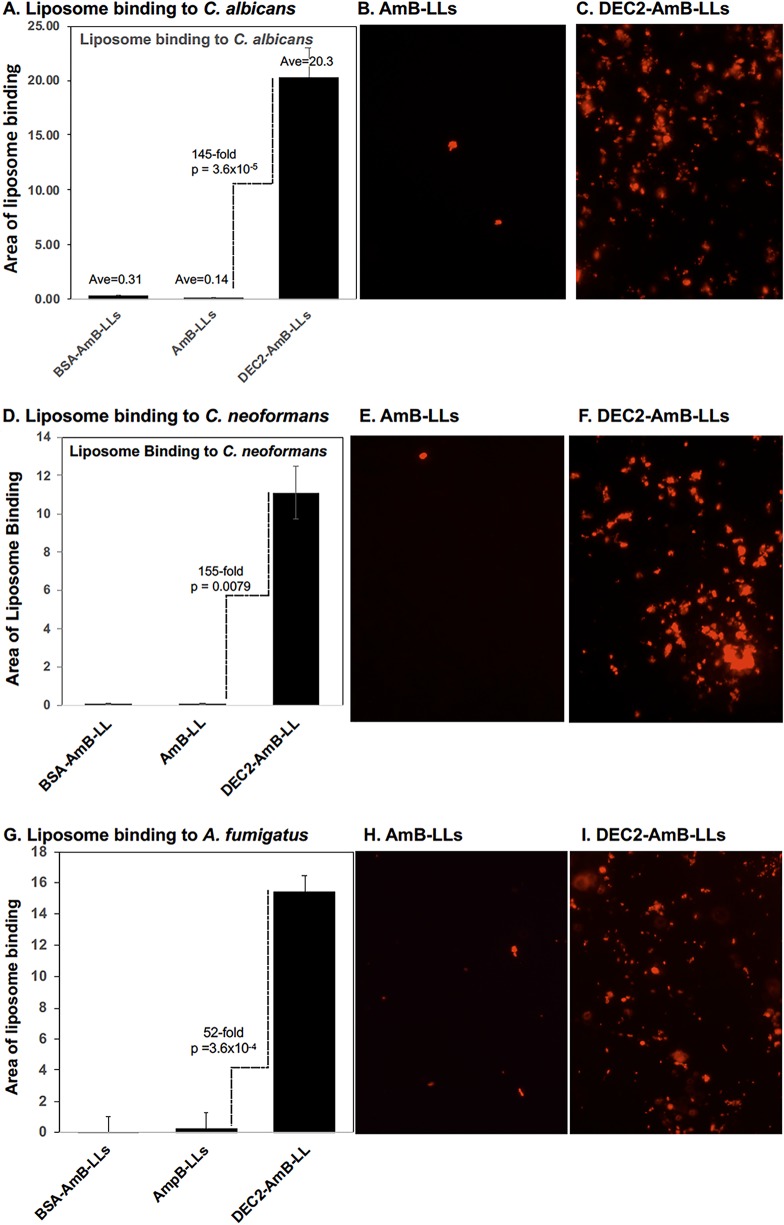
sDectin-2-coated DEC2-AmB-LLs bound 1 to 2 orders of magnitude more efficiently to C. albicans, C. neoformans, and A. fumigatus cells than control AmB-LLs. Dense fields of fixed fungal cells were incubated for 1 h with 1:200 dilutions of liposomes in liposome dilution buffer LDB2 (e.g., 0.5 μg sDectin-2/100 μl). Unbound liposomes were washed out after 1 h. Multiple fields of red fluorescent images were photographed at 20×, and the area of red fluorescence was estimated in Image J. Examples of photographic images are shown to the right of the bar graphs. (A to C) C. albicans. (D to F) C. neoformans. (G to I) A. fumigatus. In panels A, D, and G, standard errors from the mean are presented and fold differences and *P* values are indicated to distinguish the binding of DEC2-AmB-LLs from AmB-LLs.

We quantified the efficiency of DEC2-AmB-LLs binding to fungal cells compared to control uncoated AmB-LLs and BSA-coated liposomes (BSA-AmB-LLs). The area of fluorescent liposome signal was measured from multiple red fluorescent photographic images taken of liposome-stained cultures that were nearly confluent with fungal cells (Fig. 3). DEC2-AmB-LLs bound to C. albicans pseudohyphae and hyphae, C. neoformans yeast cells, and A. fumigatus hyphae 50- to 150-fold more efficiently than AmB-LLs or BSA-AmB-LLs (Fig. 3A, D, and G). Examples of the photographic images of fluorescent liposomes quantified to make these measurements are presented adjacent to each bar graph (Fig. 3B, C, E, F, H, and I).

**FIG 4:**
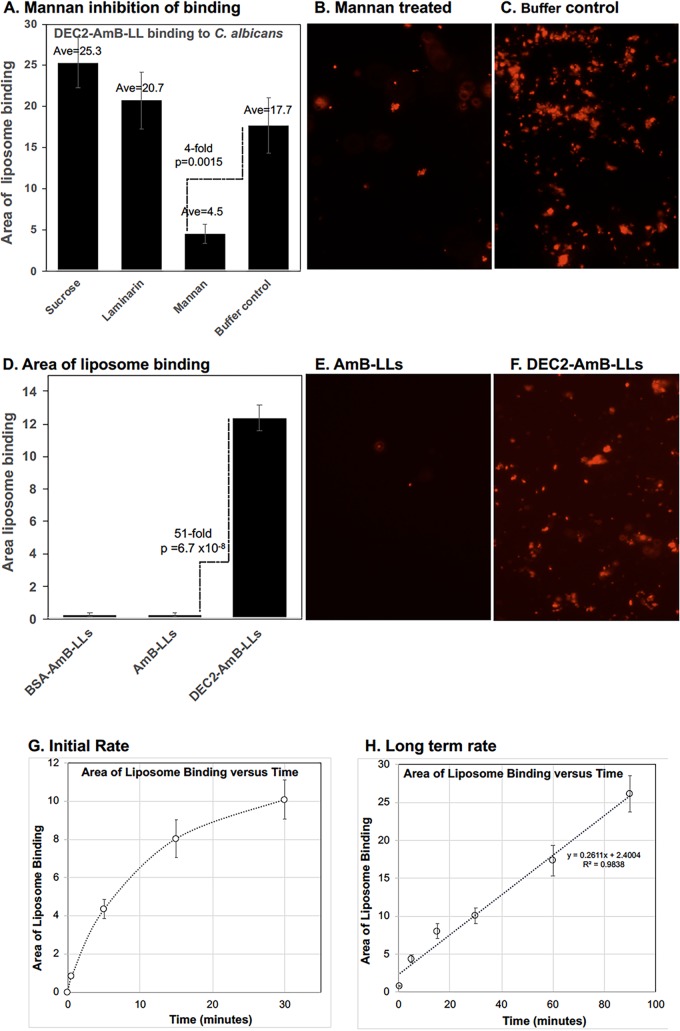
Specificity, stability, and rate of DEC2-AmB-LL binding. (A to C) Specificity of binding. DEC-AmB-LL labeling of C. albicans was inhibited by soluble yeast mannan, but not by sucrose or laminarin. Each polysaccharide was added at 10 mg/ml during a 1-h staining procedure. (D to F) Stability of binding. The plates of C. albicans cells stained with DEC2-AmB-LLs and control liposomes used to generate the data in Fig. 3A to C were left in PBS, stored in the dark for 2 months, and rephotographed, and the area of liposome binding was requantified. (G and H) Rate of binding. Mature cultures of C. albicans composed of some pseudohyphae and hyphae were grown on the surface of a 24-well microtiter plate in VMM plus 20% FBS, fixed, blocked, and treated with DEC2-AmB-LLs for the indicated times. In all three experiments, DEC2-AmB-LLs were diluted in 1:200 (wt/vol) LDB2 (0.5 μg in 100 μl sDectin-2) and washed 4 times with LDB2. Multiple red fluorescent images were taken at ×20 magnification for each time point on an inverted fluorescence microscope, and the average area of red fluorescent liposome staining was estimated. Standard errors from the mean are shown in panels A, D, G, and H. In panels A and D, the numerical average and number of fields examined are indicated above each bar and on the vertical axis. In panels A and D, fold differences and *P* values are indicated for the performance of DEC-AmB-LLs relative to mannan inhibition (A) or relative to AmB-LLs (D). These results are representative of two biological replicates.

Using the same quantitative assay of the area of fluorescent liposome binding to C. albicans pseudohyphae and hyphae, we characterized the specificity, stability, and rate of DEC2-AmB-LL binding (Fig. 4). DEC2-AmB-LL labeling was 75% inhibited by the inclusion of solubilized yeast mannans during the binding assay, but not by the same concentrations of the soluble β-glucan laminarin or of the glucose-fructose-containing disaccharide sucrose (Fig. 4A to C). This result confirmed that sDectin-2-targeted liposome binding to the extracellular matrix was mannan specific, in agreement with the published carbohydrate specificity of sDectin-2 (42–46). We examined the stability of binding of DEC2-AmB-LLs to C. albicans cells by taking the DEC2-AmB-LL-stained preparations examined in Fig. 3A and storing them in the dark in phosphate-buffered saline (PBS) at 4°C. After 2 months, these cells were rephotographed and liposomal staining quantified. The fluorescent intensity of DEC2-AmB-LLs bound to cells remained strong and was estimated to be 50-fold stronger than the nonspecific binding of AmB-LLs (Fig. 4D to F). This result suggests that the DEC2-AmB-LLs themselves are relatively stable and that their binding to cells is also relatively stable. The rate of DEC2-AmB-LL binding was estimated by exposing dense fields of C. albicans pseudohyphal and hyphal cells to liposomes for periods ranging from 10 s to 90 min before washing off unbound liposomes (Fig. 4G and H). The area of DEC2-AmB-LL labeling increased rapidly and exponentially for the first 15 min (Fig. 4G) and then slowed, but labeling did not appear to be complete after 90 min (Fig. 4H).

In summary, Dectin-2-coated AmB-loaded liposomes (DEC2-AmB-LLs) bound specifically, stably, and rapidly to fungal mannans present in the extracellular matrix of C. albicans grown *in vitro*, while little nonspecific binding of control liposomes was observed. There were only trace amounts of DEC2-AmB-LL binding to the cell wall. DEC2-AmB-LLs also bound efficiently to portions of the extracellular matrices surrounding C. neoformans and A. fumigatus cells.

### Growth inhibition and killing by DEC2-AmB-LLs.

**FIG 5:**
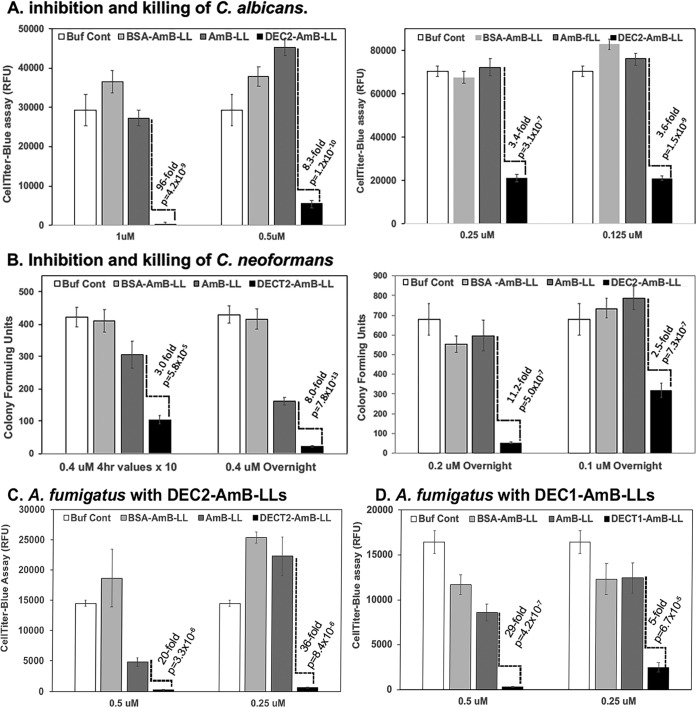
sDectin-2-coated amphotericin B-loaded liposome inhibition and killing of C. albicans, C. neoformans, and A. fumigatus. (A) C. albicans with DEC2-AmB-LLs. Cells in the pseudohyphal and early hyphal stage grown in RPMI medium plus 0.5% BSA on 96-well polystyrene microtiter plates. Cells were treated for 30 min with liposomes delivering 1.0., 0.5, 0.25, and 0.12 μM AmB to the medium as indicated, washed twice with medium, grown for an additional 16 h, and then assayed for metabolic activity using CellTiter-Blue (CTB) reagent. (B) C. neoformans with DEC2-AmB-LLs. C. neoformans cells were grown in liquid YPD medium plus 0.5% BSA with vigorous shaking for 2 h and treated for 4 h or overnight with liposomes delivering 0.4, 0.2, or 0.1 μM AmB to the medium as indicated. Cells were diluted and plated on YPD medium, and CFU were counted from multiple plates. (C) A. fumigatus with DEC2-AmB-LLs. Conidia were germinated for 9 h in VMM plus glucose plus 0.5% BSA in 96-well polystyrene microtiter plates, treated for 2 h with liposomes delivering 0.5 and 0.25 μM AmB to the medium as indicated, washed twice with medium, grown overnight, and then assayed for metabolic activity using CTB reagent in RPMI lacking phenol red indicator plus 0.5% BSA. The control wells were overgrown with hyphae protruding out the medium. (D) A. fumigatus with DEC1-AmB-LLs. Assay conditions were similar to the assay in panel C, except liposomes were first diluted into LDB1 buffer (PBS plus 0.5%BSA plus 1 mM BME) prior to dilution into growth medium. For CTB assays in panels A, C, and D, the fluorescence background from medium incubated with CTB reagent was subtracted. Standard errors are shown for all values, and fold differences and *P* values were estimated comparing the performance of AmB-LLs to DEC2-AmB-LLs. Two or more biological replicates gave similar results.

We performed various fungal cell growth and viability assays after treating actively growing cultures of C. albicans, C. neoformans, and A. fumigatus with sDectin-2-coated and control liposomes delivering AmB concentrations near the MICs for this fungicide (Fig. 5). Depending upon the assay conditions and delivery method for AmB, the MICs for AmB estimated for these species range from 0.06 to 1.3 μM (63–67). It seemed reasonable to consider that at reported AmB concentrations near the MICs for these species, we might best resolve the improved performance of targeted AmB-loaded liposomes relative to untargeted drug.

We inoculated 4,000 C. albicans yeast cells into individual wells of 96-well microtiter plates, grew them for 6 h to the pseudohyphal and early hyphal stages, and treated the cells with drug-loaded liposomes. After a 30-min incubation, liposomes were washed out and cells were grown for an additional 16 h. Figure 5A shows that targeted DEC2-AmB-LLs delivering from 1 μM down to 0.125 μM AmB killed or inhibited of C. albicans cells from 90-fold to 3-fold more efficiently than uncoated AmB-LLs or BSA-AmB-LLs delivering the same concentrations of AmB. The difference is remarkable given that in these experiments, cells were exposed to liposomal drug for only 30 min. These data were obtained using CellTiter-Blue (CTB) reagent to assess cytoplasmic reductase activity as a proxy for cell integrity and viability. Dead cells or metabolically inactive cells do not reduce the resazurin substrate to fluorescent resorufin product. Treatment of C. albicans cells continuously with liposomes for the entire 16-h time period or with higher drug concentrations resulted in too much cell death for all three drug-loaded liposome samples to clearly resolve differences among the different liposome preparations. Six-month-old preparations of DEC2-AmB-LLs retained their full antifungal activity, as long as they were freshly reduced (Materials and Methods). We also assayed viable cell numbers after liposome treatment. C. albicans yeast cells growing in rich medium in liquid culture were incubated with liposomes delivering 2 μM AmB. The liposomes were washed out after 30 min. After an additional 6 h of growth, the cultures were diluted and assayed for CFU on agar plates with rich medium. Based on CFU, DEC2-AmB-LLs were 3-fold more effective at inhibiting or killing C. albicans yeast cells in liquid than AmB-LLs or BSA-AmB-LLs (Fig. S2A).

C. neoformans yeast cells growing in liquid were treated for 4 h with liposomes delivering 0.4 μM AmB and overnight with liposomes delivering 0.4, 0.2, and 0.1 μM AmB (Fig. 5B). At the end of each treatment, cells were diluted and CFU were assayed on agar plates with rich medium. DEC2-AmB-LLs were 2.5- to 11-fold more effective at killing C. neoformans than AmB-LLs or BSA-AmB-LLs, with the optimum treatment being 0.2 μM AmB overnight. As an alternative assay, C. neoformans yeast cells growing on microtiter plates were treated for 5 h with liposomes delivering 1 μM AmB. Cells were immediately assayed for cell death by incubating them with propidium iodide. Propidium iodide enters dead but not live cells and fluoresces red when it intercalates into double-stranded DNA or short regions of double-stranded RNA. Propidium iodide assays showed that under these treatment conditions, DEC2-AmB-LLs were 5-fold more efficient at killing C. neoformans cells than uncoated AmB-LLs (Fig. S2B, C, and D).

A. fumigatus was treated with liposomes delivering AmB concentrations near and below the MIC estimated for AmBisome: 0.5 μM (67). Conidia were germinated and grown until the very early germling stage, when the germ tube first began to emerge from 95% of conidia. Cells were then treated for 2 h with AmB-containing liposomes or liposomal dilution buffer, and unbound liposomes were washed off with growth medium. Cells were grown for an additional 19 h and assayed for viability and metabolic activity with CellTiter-Blue reagent. DEC2-AmB-LLs delivering 0.5 and 0.25 μM AmB killed or inhibited the growth of A. fumigatus 20-fold and 36-fold more efficiently than AmB-LLs, respectively (Fig. 5C). It should be noted that the dilution buffer control-treated A. fumigatus cells overgrow and generate a thick hyphal mat in the microtiter wells during this assay. Hence, the metabolic activity and CellTiter Blue signal from these control cells was low.

Dectin-1-targeted AmB-loaded liposomes also bind efficiently to A. fumigatus swollen conidia, germlings, and hyphal cells and inhibit and kill these cells (51), although they are binding β-glucans and not α-mannans. In this previous study, cells were incubated continuously with liposomes during the entire assay and not washed out. For a more direct comparison of the drug-targeting efficiency of the two Dectins, we examined DEC1-AmB-LLs using the same assay design used herein for DEC2-AmB-LLs, washing out the liposomes after 2 h, except that liposomes were initially diluted into LDB1 and the cells were grown out for 16 h (see Materials and Methods). DEC1-AmB-LLs delivering 0.5 and 0.25 μM AmB killed or inhibited the growth of A. fumigatus 28-fold and 5-fold more efficiently than AmB-LLs, respectively (Fig. 5D). Using this assay condition, the results from AmB-loaded liposomes targeted by Dectin-1 and Dectin-2 are very similar.

### Toxicity of DEC2-AmB-LLs to animal cells.

Rapidly grown cultures of human HEK-293 and human HT-29 cells were treated overnight with various liposomes and a deoxycholate micelle suspension each delivering 15 μM AmB (AmB/micelles). Based on CellTiter-Blue assay of metabolic activity and viability, DEC2-AmB-LLs were 10% to 20% more toxic than AmB-LLs or BSA-AmB-LLs and 2- to 5-fold less toxic than AmB deoxycholate (AmB/DOC) micelles (Fig. S3). When these cells were treated to receive lower AmB concentrations (for example, 3 μM AmB), only AmB/DOC micelles showed measurable toxicity. None of the three liposomal preparations appeared to have any particular affinity for either of these cell lines when examined by fluorescence microscopy.

## DISCUSSION

We coupled the N-terminal domain of Dectin-2, sDectin-2, to a lipid carrier and inserted this conjugate into liposomes such that the C-terminal carbohydrate recognition domain (CRD) of each monomer faced outward from the liposomal membrane. Considering the efficient binding we obsered for these DEC2-AmB-LLs to the extracellular matrices of three diverse human fungal pathogens, the sDectin-2 monomers must have been conformationally free to form the functional dimers necessary for efficient mannan binding (48, 68). C-type lectin receptors often bind some of their substrates weakly. The published estimated effective concentrations for 50% of sDectin-2 binding to mannan-related polysaccharides (EC_50_s) range from approximately 20 mM for mannose and 2 mM for mannan-α-1-2-mannan (68) down to 150 μM for mannoglycan (44, 68). This is indeed weak binding compared to Dectin-2’s closest paralog, Dectin-1, which has EC_50_s for binding various β-glucans ranging from 2 mM down to 2.2 pM (69). It is likely that the greater avidity created by having approximately 1,500 sDectin-2 monomers on each liposome resulted in the rapid, strong, and stable binding we oberved for DEC2-AmB-LL binding to fungal cells.

Most pathogenic fungal species, including *Candida*, *Cryptococcus*, and *Aspergillus*, live within polysaccharide matrices that promote adherence to host cells, tissues, and implanted biomedical devices, and which partially protect them from host antifungal ativities (70–73). The exopolysacchride matrix of C. albicans contains approximately 13% β-1,6-glucan and 87% α-1,6-mannan (74). C. neoformans produces a glucuronoxylomannan- and galactoxylomannan-rich capsule (75) that is shed and contributes to formation of the extracellular matrix. The matrix is composed of 20% mannan and 23% glucan residues (76). The exopolysaccharide matrix of A. fumigatus contains approximately 14% galactomannan and 13% β-glucans (77). Our data on the efficient binding of DEC2-AmB-LLs to the extracellular matrix of all three species is consistent with the mannan-rich content of their exopolysaccharides. However, some regions of the C. albicans, C. neoformans, and A. fumigatus matrices did not stain or stained poorly with sDectin-2-coated liposomes, suggesting that either the distribution of mannans in the matrix is heterogeneous or the mannans in these regions are masked from exposure to liposomal sDectin-2. This distinction between mannan exposure or expression within their exopolysaccharide matrices remains to be resolved. We showed that the binding of DEC2-AmB-LLs to exopolysaccharide mannans was no more restricted than the binding of the much smaller DEC2-Rhod, suggesting size restriction may not be the primary limitation.

We did not find convincing evidence that DEC2-AmB-LLs bound more than at trace levels to the cell walls of any of these fungal species. Although the cell wall mannan content varies widely based on growth media and methods of chemical analysis, the estimated cell wall mannan polysaccharide content of C. neoformans is 22% (78), that of C. albicans is 40% (79), and that of A. fumigatus is 15% to 41% (73, 79). Although rhodamine-labeled sDectin-2 (DEC2-Rhod) bound with similar intensity and pattern to the exopolysaccharide matrix of A. fumigatus as DEC2-AmB-LLs, DEC2-Rhod did not stain the cell wall. Hence, again a size barrier for the much larger DEC2-AmB-LLs does not appear to explain their lack of cell wall binding. Hence, our findings suggest the cell wall mannans may be chemically masked from sDectin-2-mediated liposomal binding. This result parallels the masking of C. albicans cell wall β-glucans from sDectin-1 binding and from DEC1-AmB-LL binding (52, 54–56).

C. albicans reversibly switches among unicellular ovoid yeast and multicellular ellipsoid pseudohyphal and elongated hyphal morphologies (80). All three stages are capable of producing an extracellular matrix and adhering to host tissues. The matrices of all three bound DEC2-AmB-LLs, suggesting fungicide-loaded Dectin-2-coated liposomal therapeutics have the potential to reduce the virulence of C. albicans.

DEC2-AmB-LLs inhibited and killed C. albicans, C. neoformans, and A. fumigatus far more efficiently than plain uncoated AmB-LLs or BSA-AmB-LLs delivering the same concentrations of AmB. A combination of metabolic activity assays based on CellTiter-Blue (CTB) reagent, cell growth assays based on CFU, and propidium iodide staining of dead cells confirmed that both inhibition and killing of cells occurred. Incubation with DEC2-AmB-LLs for as little as 30 min to a few hours resulted in significant killing. DEC2-AmB-LLs were 3- to 90-fold more efficient at inhibiting or killing fungal cells, when delivering AmB concentrations near or below the MIC values reported for AmB, concentrations at which our AmBisome equivalent uncoated AmB-LLs had little or no impact on cell inhibition or survival. The DEC2-AmB-LLs bound all three species 50- to 150-fold better than AmB-LLs and, under some conditions we tested, inhibited and killed them 11- to 94-fold more efficiently than AmB-LLs. We believe the differences in the optimal results for the binding and killing assays and the variability within the killing assays are due primarily to the fact that inhibition and killing assays are much more complex and difficult to optimize. They are influenced by many variables, including (i) the amount of AmB being delivered, (ii) the cells proceeding through more than one developmental stage during the assay, (iii) the time of liposome exposure, (iv) the length of the growth period after liposome exposure, and (v) the mechanics of the assay being used. The binding assays are influenced primarily by the concentration of sDectin-2 and the extent of washing.

A significant reduction in the MIC for AmB or other liposomal packaged therapeutic fungicides should result in reducing fungicide doses and the frequency of drug administration, which, in turn, should reduce host toxicity. We showed that DEC2-AmB-LLs were not particularly toxic to animal cells when delivering 15 μM AmB, which was 15- to 150-fold higher than the AmB concentrations used here to kill fungal cells. However, there are number of important factors that we have not yet explored *in vitro*, which may alter the amount of exopolysaccharide matrix and its composition and may influence the effectiveness of Dectin-2-targeted antifungals *in vivo*. These include growth substrate, temperature, carbon source, serum, and the particular antifungals being delivered.

*Candida*, *Cryptococcus*, and *Aspergillus* species belong to three evolutionarily disparate classes of fungi (81), the Saccharomycetes (phylum Ascomycota), Tremellomycetes (phylum Basidiomycota), and Eurotiomycetes (phylum Ascomycota), respectively. It is estimated that they separated from common ancestry relatively early in the evolution of the fungal kingdom, 0.8 to 1.3 billion years ago (82). DEC2-AmB-LLs bound specifically to the extracellular matrix of all three. This suggests the mannans found in the extracellular matrices of most pathogenic fungi will be conserved enough in structure to bind sDectin-2-targeted liposomes. However, mannan expression and masking from host detection may vary widely among pathogenic fungal species or in different host niches. Consistent with the masking of mannans to sDectin-2 binding, our assays showed very inefficient binding to the mannan-rich cell walls of all three species.

This preliminary study was performed with the mouse sDectin-2 protein sequence to avoid problems of sDectin-2 immunogenicity during the future testing of sDectin-2-targeted antifungals in mouse models of candidiasis, aspergillosis, and cryptococcosis. The human sDectin-2 protein sequence is 72% identical to the mouse protein and is only 2 amino acids (aa) shorter. Therefore, we do not expect problems manipulating the human protein to target fungicide-loaded therapeutic liposomes for use in clinical studies.

In summary, there is a dire need for new antifungal therapeutics. DEC2-AmB-LLs bound efficiently to the extracellular matrices produced by diverse cellular stages of C. albicans, C. neoformans, and A. fumigatus. DEC2-AmB-LLs delivering AmB concentrations near or below AmB’s MICs for growth inhibition and killing of all three species showed sDectin-2 targeting of the liposome-packaged drug improved the fungicidal effect by an order of magnitude or more over untargeted liposomal AmB. It is reasonable to propose that, drug-loaded liposomes targeted to fungal cells have significant potential as pan-antifungal therapeutics with a wide range of applications. The next step in our research will be to examine the efficacy of targeted liposomal antifungal drugs in mouse models of invasive fungal diseases.

## MATERIALS AND METHODS

### Cell culture.

C. albicans CAI4 expressing green fluorescent protein (GFP) under the control of the *ADH1* promoter (83), A. fumigatus A1163 (51), and wild-type C. neoformans H99-alpha (84) were grown in Vogel’s minimal medium (VMM) plus 1% glucose (85) plus 0.5% BSA or RPMI 1640 medium with no red indicator dye (Thermo Fisher SKU-11835-030) plus 0.5% BSA or YPD (1% yeast extract, 2% peptone, 2% dextrose) in liquid with shaking or on 24- or 96-well polystyrene microtiter plates or on glass microscope chamber slides and incubated at 37°C for 3 to 36 h. Plates were precoated with poly-l-lysine for A. fumigatus, and glass microscope slides were precoated with for all three species. All fungal cell growth was carried out in a biosafety level 2 (BSL2) laboratory. Before cells were treated with fluorescent liposomes for microscopic analysis of binding, fungal cells were washed three times with PBS, fixed in 4% formaldehyde in PBS for 60 min, washed three times, and stored at 4°C in PBS.

The human colorectal adenocarcinoma cell line HT-29 (ATCC HTB-38) and human embryonic kidney cell line HEK-293 (ATCC CRL-1573) were grown in 96-well microtiter plates in RPMI medium lacking red indicator dye plus 10% fetal calf serum in a 37°C incubator in air supplemented with 5% CO_2_. Their viability and metabolic activity after overnight antifungal treatment were assayed using CTB reagent diluted 1:10 into the medium and with incubation for 60 to 90 min at 37°C with 8 wells per treatment.

### Production and chemical modification of sDectin-2.

The carboxy-terminal end of Dectin-2 contains its mannan recognition domain, sDectin-2. The sequence of the codon-optimized E. coli expression construct with MmsDectin-2lyshis synthesized and cloned into pET-45B by GenScript is shown in Fig. S2. The 577-bp-long DNA sequence encodes a slightly modified 189-amino-acid (aa)-long sDectin-2 protein containing a vector-specified N-terminal (His_6_) affinity tag, an additional flexible GlySer spacer, the sequence LysGlyLys with lysine residues for cross-linking, another flexible spacer followed by the C-terminal 166-aa-long murine sDectin-2 domain. The modified sDectin-2 protein, DEC2 was expressed in E. coli and purified as described previously for mouse sDectin-1 (51) followed by an extra step of gel exclusion chromatography on Sephacryl S-100 HR (GE Healthcare, no. 17061210). The protein is shown on an SDS-PAGE gel stained with Coomassie blue in Fig. S3. Samples of sDectin-2 at 5 μg/μl in the same guanidine hydrochloride (GuHCl) buffer with freshly added 5 mM β-mercaptoethanol (BME) were adjusted to pH 8.3 with 1 M pH 10 triethanolamine and reacted with the reactive succinimidyl ester NHS moiety of a 4 M excess of DSPE-PEG-3400-NHS (1,2-distearoyl-*sn*-glycero-3-phosphoethanolamine [DSPE]-conjugated polyethylene glycol [PEG], from Nanosoft Polymers; no. 1544-3400) for 1 h at 23°C to make DEC2-PEG-DSPE (Fig. S1). Size exclusion chromatography through Bio-Gel P-6 acrylamide resin (Bio-Rad no. 150-0740) in renaturation and storage buffer RN#5 (0.1 M NaH_2_PO_4_, 10 mM triethanolamine, pH 8.0, 1 M l-arginine, 100 mM NaCl, 5 mM EDTA, 5 mM β-mercaptoethanol) removed unincorporated DSPE-PEG and GuHCl (51). BSA-PEG-DSPE was prepared from BSA (bovine serum albumin; Sigma, A-8022) by the same protocol, but with buffer lacking the GuHCl during DSPE-PEG labeling and l-arginine during Bio-Gel P6 chromatography. Rhodamine-labeled sDectin-2 (DEC2-Rhod) was prepared by the same procedure used to make DEC2-PEG-DSPE in the same GuHCl buffer, but labeling was performed with a 4 M excess of rhodamine-NHS reagent (Thermo Fisher no. 46406) over sDectin-2. Hydrolyzed unbound rhodamine reagent and unwanted salts were removed from DEC2-Rhod using size exclusion chromatography on Bio-Gel P2 resin in RN#5 buffer.

### Remote loading of AmB, sDectin-1, BSA, and rhodamine into liposomes.

Starting with sterile pegylated liposomes from FormuMax Scientific, Inc. (DSPC:CHOL:mPEG2000-DSPE; FormuMax no. F10203A), we prepared small batches of liposomes with 11 mol% AmB (Sigma A4888) relative to 100% liposomal lipid to make AmB-LLs as described previously (51) (Table S1).

The DEC2-PEG-DSPEand BSA-PEG-DSPE conjugates in RN#5 buffer and PBS, respectively, were integrated via their lipid DSPE moiety into the phospholipid bilayer membrane of AmB-LLs by 30 min of incubation at 60°C to make DEC2-AmB-LLs and BSA-AmB-LLs, parallel to the protocol used previously (51). During these same 60°C incubations, 2 mol% of the red fluorescent tag Lissamine rhodamine B-DHPE (Invitrogen, no. L1392) was also incorporated into sDectin-2- and BSA-coated liposomes and AmB-LLs (51). A duplicate sample of the DEC2-AmB-LLs was subjected to gel exclusion chromatography over 0.5 M Bio-Gel A resin (Bio-Rad, no. 151-0140). Fluorescent liposomes were efficiently excluded from the resin. Because no sDectin-2 (optical density at 280 nm [OD_280_] of 2.2/mg/ml) or rhodamine was detected in the low-molecular-weight fractions included in the gel, we concluded that both were efficiently loaded into liposomes. Fresh 2 mM BME was added to DEC2-AmB-LLs before each use in binding or killing assays. DEC2-AmB-LLs stored at 4°C in RN#5 appeared to retain full fungal cell binding specificity and killing activity for at least 12 months.

### Microscopy of liposomes and DEC2-Rhod bound to fungal cells.

Formalin-fixed fungal cells were incubated with liposomes at 23°C in liposome dilution buffer LDB2 (20 mM HEPES, 10 mM triethanolamine, 150 mM NaCl, 10 mM CaCl_2_, 1 mM BME, 5% BSA, pH 8.0), wherein the BME was added fresh. Liposomal stocks were diluted 1:200 before incubation with cells such that the sDectin-2 protein concentration was 0.5 μg/100 μl. After incubations of 15 min, 1-h, or longer, unbound liposomes washed out with 4 changes of LDB2. Merged images of rhodamine red fluorescent liposomes, green fluorescent cells, and differential interference contrast (DIC) illuminated cells were taken of cells grown on microscope slides under oil immersion at 63× on a Leica DM6000B automated microscope. The DEC2-Rhod stock was also diluted 1:200 before incubation with cells such that the sDectin-2 protein concentration was also 0.5 μg/100 μl. Bright-field, DIC, and red (excitation of 560 and emission of 645 [Ex560/Em645]) and green (Ex500/Em535) fluorescent images were taken of cells on microtiter plates at 20× on an Olympus IX70 inverted microscope using an Olympus PEN E-PL7 digital camera, and the bright-field and/or fluorescent-colored layers were merged in Photoshop. The area of liposome binding at ×20 magnification was quantified by taking an 8-bit grayscale copy of the unmodified red fluorescent TIF image into Image J (imagej.nih.gov/ij), using Image>Adjust>Threshold>Apply to capture just the red fluorescent areas illuminated by liposomes, and using Analyze>Measure to place the area data for each image in a file. Six to 10 photographic images were generally analyzed and averaged for most area estimates. However, because the staining intensities varied widely among photographic fields of C. neoformans cells, 90 images were analyzed for each treatment. Bright-field, DIC, and fluorescent images were also taken of cells grown in microsope chamber slides at 20× or under oil immersion at 63× on a Leica DM6000B automated microscope.

The glucuronoxylomannan (GXM)-specific monoclonal antibody 18B7 (60, 61) was obtained from Sigma-Aldrich (MABF2069), used at a 1:200 dilution (0.5 μg/100 μl), and visualized with secondary goat anti-mouse antibody Alexa 488 (Life Technologies, no. A11001) also diluted 1:200 and photographed with GFP filters (Ex500/Em535).

### Growth inhibition and viability assays following liposome treatment.

Liposomal stocks were stored at 615 to 800 μM AmB and typically diluted first 30- to 600-fold into liposome dilution buffer, LDB2, and then diluted 1:11 into growth medium to achieve the indicated final fungicide concentrations ranging from 2 μM down to 0.1 μM. Control cells received an equivalent amount of LDB2. CellTiter-Blue (CTB) cell viability and metabolic activity assays of C. albicans and A. fumigatus were conducted as we recently described for A. fumigatus, by incubating cells with CTB reagent for 3 to 4 h (51) and analyzing 96-well plates in a Bio-Tek Synergy HT fluorescent microtiter plate reader. The fluorescent background from control wells was subtracted from experimental wells. Data from eight wells were averaged for each data point. These assays had a lot of background and were less sensitive if the cells were assayed in VMM. We were unable to detect any fluorescent signal from CTB reagent when performing parallel cell viability assays on C. neoformans using this protocol or another CTB protocol published for this species (86). As an alternative measure of C. neoformans and C. albicans cell viability, assays were conducted by growing 1 ml of cells in YPD, adding drug-loaded liposomes for the indicated times of growth, diluting the cells, plating on YPD, and counting CFU. The fraction of dead C. neoformans cells among cells growing in YPD after treatment with fungicide-loaded liposomes was assayed by adding propidium iodide to the medium at 50 μg/ml and incubating for 60 min at 37°C. The medium was removed and replaced with PBS for fluorescence microscopy using the red fluorescent protein channel (Ex560/Em595) and scoring the percentage of dead stained cells relative to the total number of stained and unstained cells. In experiments with DEC1-AmB-LLs, all liposomes were diluted with LDB1 (PBS plus 5% BSA plus 1 mM BME) (51) instead of LDB2.

10.1128/mSphere.00715-19.5 FIG S5 DEC2-AmB-LL killing of C. albicans *and* C. neoformans relative to control liposomes. (A) C. albicans yeast cells treated in liquid culture and assayed for viability. C. albicans yeast cells growing in liquid culture in YPD were treated for 30 min with amphotericin B-loaded liposomes delivering 2 μM amphotericin B. Cells were diluted immediately after treatment and plated on YPD agar plates, and CFU were averaged from nine separate assays of fungal colony numbers for each treatment. The buffer control cells received an equivalent amount of liposome dilution buffer LDB2, but no liposomes. (B to D) C. neoformans yeast cells treated in liquid culture and assayed for viability. Each well of a 96-well microtiter plate was seeded with 1,000 C. neoformans cells in YPD medium, grown for 5 h, treated for 4 h with liposomes delivering 1 μM AmB or liposome dilution buffer (LDB2), washed once, and stained for 30 min with propidium iodide. The total cell number and the number of propidium-positive red fluorescent cells (dead cells) were counted manually from 10 photographic fields taken at 20× magnification. The percentage of cells scored as dead is indicated for each treatment. The standard errors are shown along with the fold difference and a *P* value comparing the performance of AmB-LLs to DEC2-AmB-LLs. Download FIG S5, JPG file, 0.8 MB. Copyright © 2019 Ambati et al. 2019 Ambati et al. This content is distributed under the terms of the Creative Commons Attribution 4.0 International license.

10.1128/mSphere.00715-19.6 FIG S6 Toxicity of sDectin-2-coated DEC-AmB-LLs to human cells. (Left) Human HEK-293 cells. (Right) Human HT-29 cells. Cells in 96-well microtiter plates were grown to 30% confluence and treated overnight with various AmB-containing liposomes and an AmB/DOC micelle suspension, each delivering 15 μM AmB to the medium. The buffer control wells received an equivalent amount of liposome dilution buffer LDB2. CellTiter-Blue assays estimated cell viability and survival. Background fluorescence from wells with CellTiter-Blue reagent in the medium, but lacking cells and liposomes, was subtracted. Standard errors are indicated. Fold difference and *P* values are indicated for comparisons of the performance of AmB/DOC and AmB-LLs to that of DEC2-AmB-LLs. For this experiment, we prepared an AmB-DOC (deoxycholate) micelle stock suspension (4,320 μM AmB), parallel to commercial preparations known as AmBd by vortexing 2.3 mg AmB (2.5 μmol) plus 3.8 mg sodium DOC (9.1 μmol) in 0.5 ml 40 mM phosphate buffer (pH 7.4), heating for 10 min at 60°C, and homogenizing for 2 min (88, 89). Their composition is shown in detail in Table S1. Download FIG S6, JPG file, 0.6 MB. Copyright © 2019 Ambati et al. 2019 Ambati et al. This content is distributed under the terms of the Creative Commons Attribution 4.0 International license.
