# Supplementary figures and images for: Dectin-2-Targeted Antifungal Liposomes Exhibit Enhanced Efficacy
Source: mSphere. 2019 Oct 30;4(5):e00715-19. doi: 10.1128/mSphere.00715-19 (PMC6821932; doi:10.1128/mSphere.00715-19)

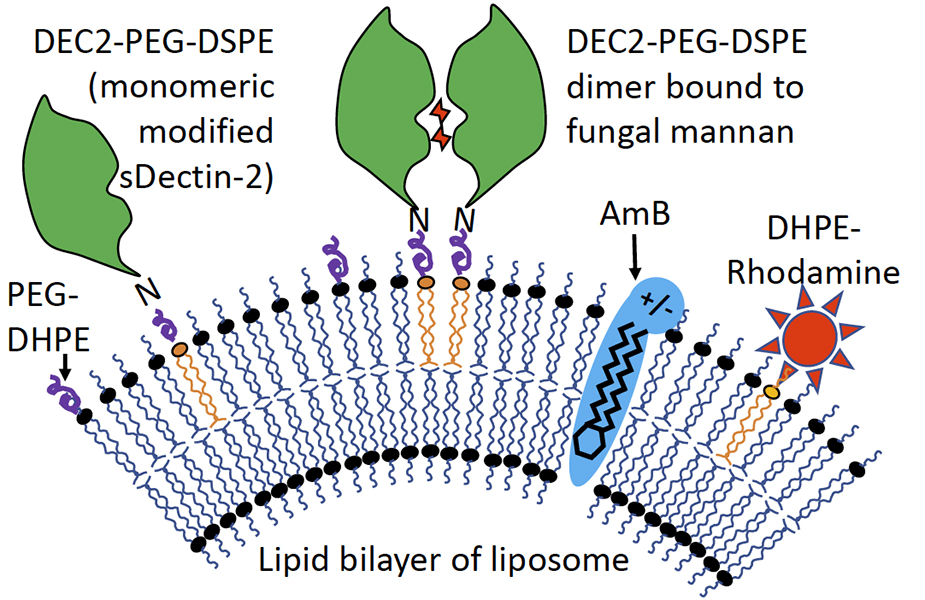

Supplement: FIG S1 [file mSphere.00715-19-sf001.jpg]

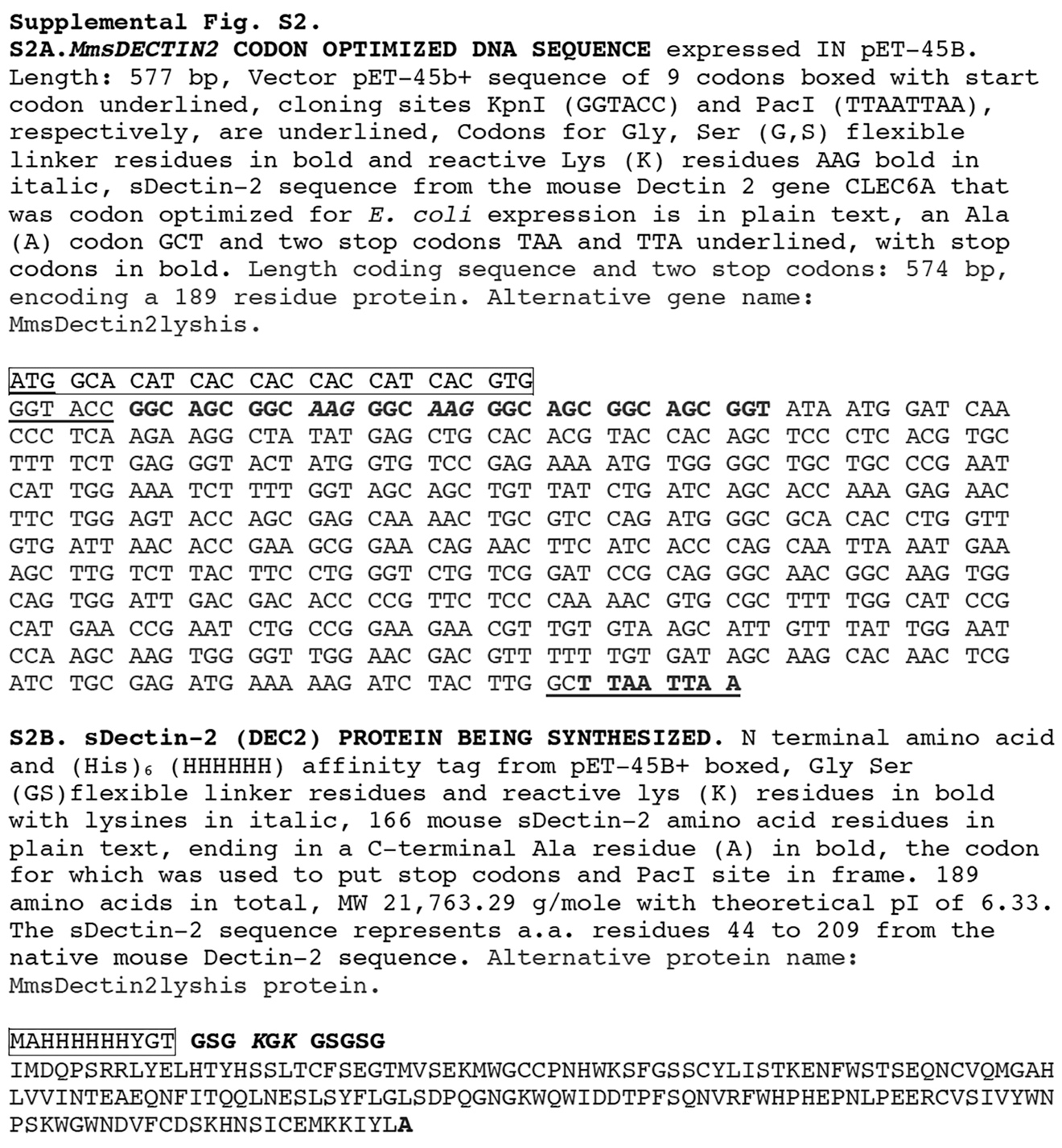

Supplement: FIG S2 [file mSphere.00715-19-sf002.jpg]

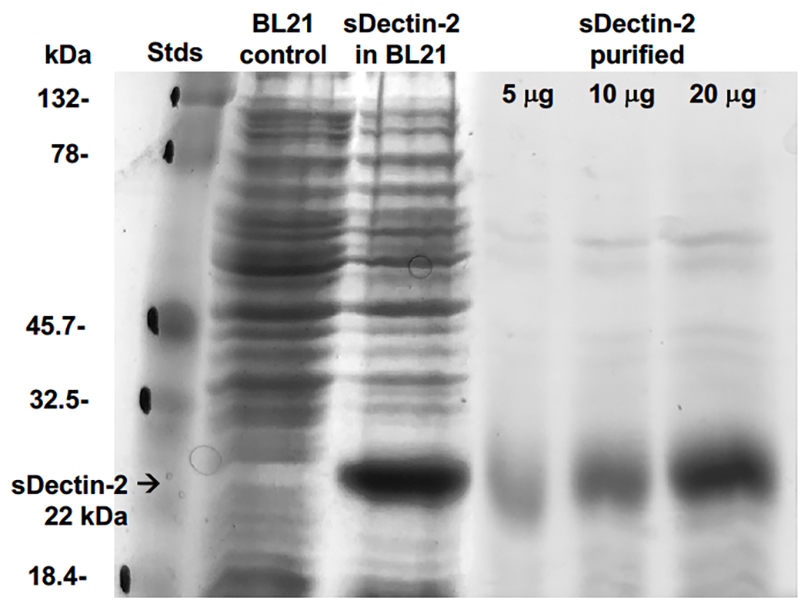

Supplement: FIG S3 [file mSphere.00715-19-sf003.jpg]

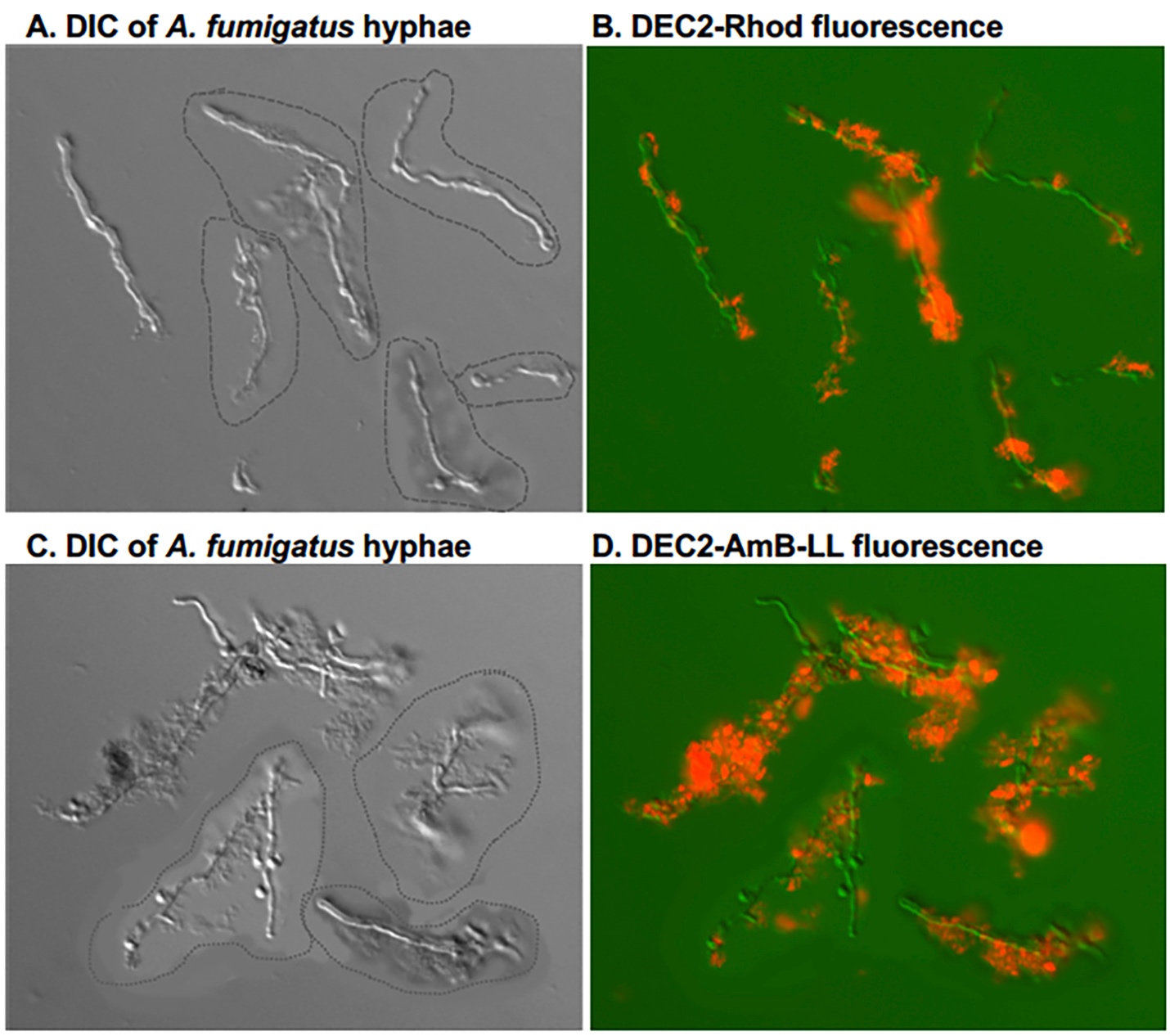

Supplement: FIG S4 [file mSphere.00715-19-sf004.jpg]

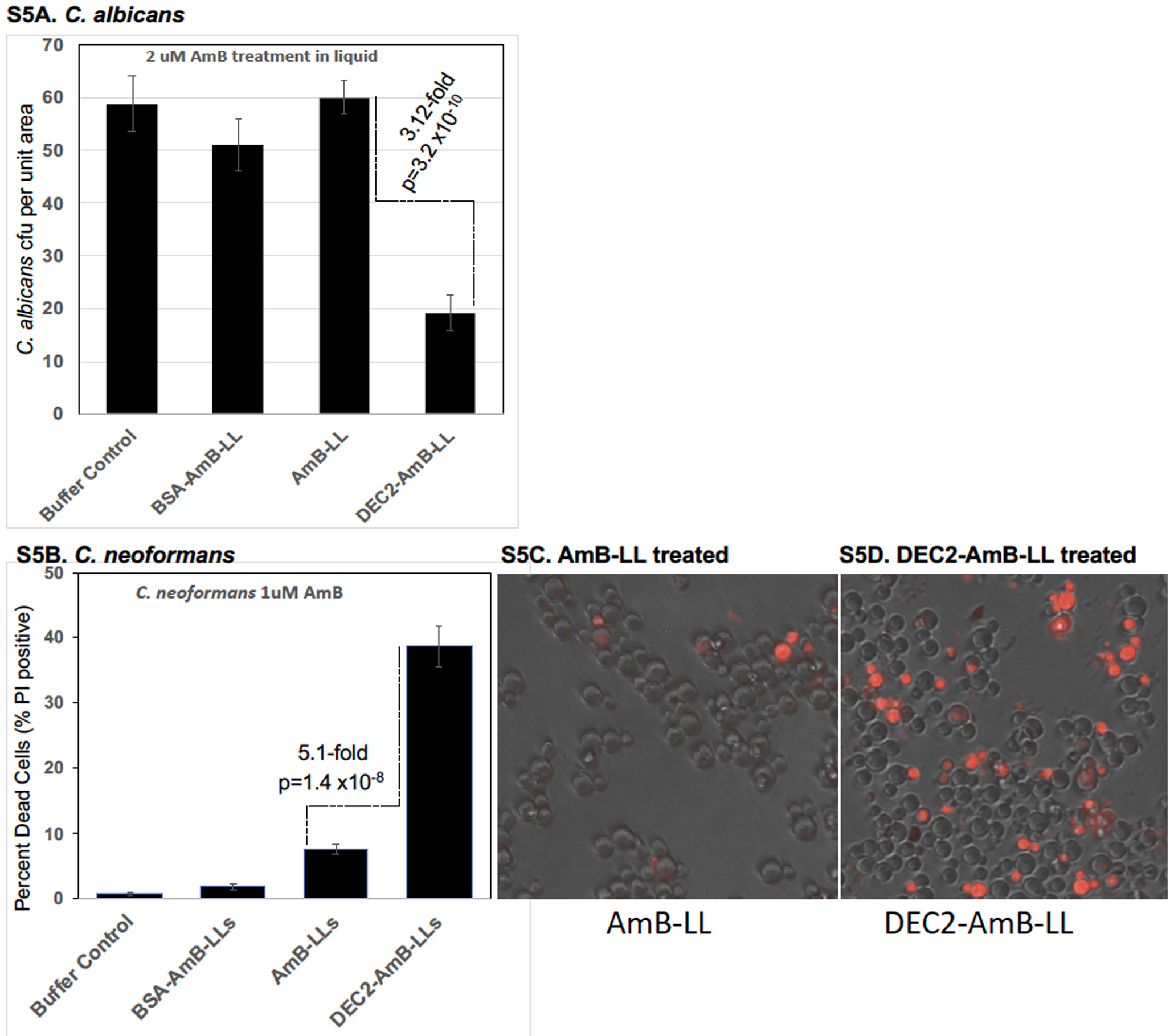

Supplement: FIG S5 [file mSphere.00715-19-sf005.jpg]

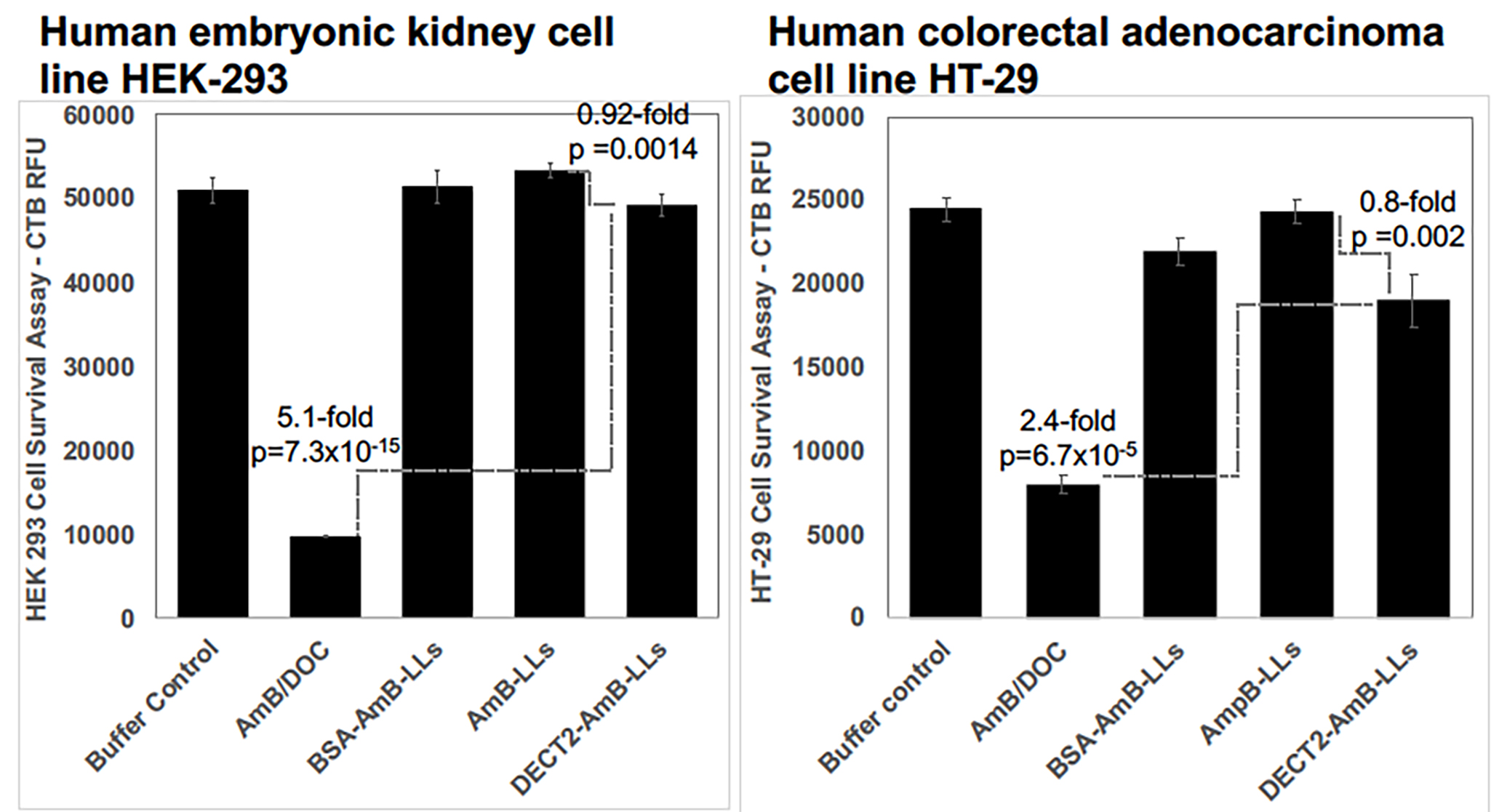

Supplement: FIG S6 [file mSphere.00715-19-sf006.jpg]
